# Therapeutic Vaccines: Hope Therapy and Its Effects on Psychiatric Symptoms among Infertile Women

**DOI:** 10.5539/gjhs.v6n1p192

**Published:** 2013-11-25

**Authors:** Leili Mosalanejad, Khadije Abdolahifard, Masoumeh Golestan Jahromi

**Affiliations:** 1 Mental health Department, Jahrom University of Medical Sciences, Jahrom, Iran; 2 Research Department, Jahrom University of Medical Sciences, Jahrom, Iran; 3 Research and clinical center for infertility, ShahidSadoughi University of Medical Sciences, Yazd, Iran

**Keywords:** hope therapy, depression, anxiety, stress, infertility

## Abstract

Infertility is a life crisis which leads to serious psychological problems. The present study aims to investigate the effects of hope therapy as a psychological intervention on psychological distresses among infertile women.

The present study was an experimental one. The study population included infertile women referring to gynecology clinics. Women who lived in Jahrom and could take part in psychotherapy sessions, had no chronic physical or mental disorders, suffered from primary infertility, had infertility unknown causes and had no history of miscarriage and stillbirth were selected through convenience sampling method and were divided into control and intervention groups (n=61). Women in the intervention group participated in eight 2-hour sessions for a period of 2 months.

Study results revealed that there was a significant difference between the two groups after the intervention. Besides, there was a significant difference between the two groups through paired T-test (p<0.05). Furthermore, results of ANCOVA showed that after eliminating demographic variables, the intervention was effective in the total mean difference of the study groups. It means that the difference between the two groups was resulted from intervention.

Hope therapy as a positive psychological approach can improve infertile women’s general health and subsequently improve family’s health. Therefore, in addition to assisted reproductive techniques, hope therapy is recommended to be presented to infertile people in order to improve the quality of their life and help them adapt with their problems.

## 1. Introduction

Infertility is a life crisis which leads to serious psychological problems and stressful experiences for the people (Sadock et al., 2003). After mother’s death, father’s death and spouse betrayal, infertility is the fourth stressful event of life (Oddens et al., 2010). Infertility is a stressful event in life and reproductive failure is quite important regarding its cultural dimension. A large number of infertile couples encounter repeated painful treatment courses without any outcomes which can cause a feeling of weakness in achieving one’s aim (van den Akker, 2005). Overall, infertility and its treatment are very difficult and are accompanied by the experience of chronic stress. It is also associated with the increase in the risk of symptoms and psychological problems (Schmidt & Sejbæk, 2005).

Group therapy techniques (psychoanalysis, cognitive, behavioral) not only are effective in prevention and treatment of various psychological techniques such as anxiety, stress, and phobia, but also play a key role in physical health and infertility rate (Ramezanzadeh et al., 2007).

Studies on the effect of psychological and consultative interventions on the psychological disorders and pregnancy outcomes in infertile couples have shown that psychological interventions have been effective in reducing anxiety and depression and increasing pregnancy rate (Domar et al., 1999). In the same line, Kupka et al. (2003) reported that psychological interventions led to spontaneous pregnancy in 14% of the cases which might have resulted from treatment of stress.

Nowadays, different approaches are being used for improving psychological health and decreasing psychological distresses in infertile women. In spite of the fact that nowadays people have their required facilities to live their lives, they have not got any sense regarding how to live. In fact, technology has deprived us from the need to use survival skills (Pippin, 1997).

In general, infertile people suffer from various psychological problems of which hopelessness is one of the main outcomes. Lack of hope for having a child which is highly valuable for a woman is accompanied by a great grief that is even intensified by seeing other mothers and children around. This causes a feeling of lack of self-control or self-confidence eventually leading to reduction of self-esteem. In fact, infertility completely disturbs a woman (Homan et al., 2007). Such women experience anxiety, depression, lack of control, shame, and low self-confidence; such feelings have been reported even up to 18 months after unsuccessful IVF (Klonoff-Cohen, 2005). Women in reproductive ages spend a great energy on having a child and it is not surprising that they feel depressed and worried after months of unsuccessful attempts (American Society for Reproductive Medicine 2007).

Hope is one of the main psychological needs of human. Hope for future provides people with motivation, vitality and exhilaration and is also associated with philosophy of life. In other words, hope leads to a feeling of self-confidence and a positive internal feeling toward a particular phenomenon. In addition, hope reduces fear, anxiety, and fatigue and gives meaning to the people’s deeds. It is in fact impossible to achieve a goal without hope. On the contrary, hopeful people believe that the darkest scenario will not happen to them (Takhavi, 2010). Hope predicts physical and mental health identified by indexes, such as self-reported health, positive response to medical interventions, mental health, positive mood, immunological strength, effective coping, re-evaluation, problem solving, avoiding stressful life events, seeking for support, and health improving behaviors (Felder, 2000). Using Snyder’s theory, the effect of hope has also been assessed on the process of death in final stages of diseases. In this theory, hope has been identified as the capability to understand the practical ways for reaching goals and the necessary thought and motivation for using these strategies. Accordingly, maintaining and increasing the dying hope of patients is quite beneficial in medical interventions (Peterson, 2000). In fact, it helps clients to formulate their goals, consider different paths for reaching their goals, motivate themselves to follow their goals, and consider barriers as challenges to be overcome. The founder of hope and hope therapy has considered hope as a type of treatment (Gum & Snyder, 2002). Some other researchers have stated hope to play a critical role in people’s attitude toward their disease (Takhavi, 2010). Overall, psychotherapists all over the world are investigating hope as an important experimental belief. In comparison to false hope (optimism), real hope (hopefulness) leads to positive health, while the decrease in hope is accompanied by weak results. Thus, psychologists have to discover hope and belief issues in patients in order to facilitate the treatment process during a life crisis. In fact, this can be an important part of treatment of a depressed patient (Snyder, 2002).

Studies conducted on optimism and hopefulness have shown that in comparison with pessimist people, optimist ones experience less physical weakness, depression, and hopelessness and are less likely to commit suicide in case of being faced with stressful life events(Snyder, 2002; Clarke, 2003).

Considering the fact that fertility is an important subject in Iranian cultural context and since psychological approaches highly affect patients’ mental problems and psychological treatments can be effective in improving patients’ health, steps should be taken toward providing the ground for reducing psychological problems of infertile people and improving their mental health. Up to now, no studies have been conducted on the effect of hope therapy on infertile women. Therefore, the present study aims to investigate the effect of hope therapy on psychological problems (anxiety, depression, and stress) of infertile women who were being treated by assisted reproductive techniques.

## 2. Materials and Methods

The present study was an experimental one. The study population included all infertile women referring to gynecology clinics affiliated to Jahrom University of Medical Sciences, south east of Iran during 6 months. Women who lived in Jahrom and could take part in psychotherapy sessions, unknown cause, those who had no chronic physical or mental disorders, women who suffered from primary infertility, infertility unknown causes and had no history of miscarriage and stillbirth were selected through convenience sampling. 10 patients were excluded because they did not participate in regular sessions and did not complete the questionnaire. So the study was continued by 61 women. Women were then divided into intervention (n= 30) and control group (n=31). Two women were absent in two sessions and were excluded from the study. All the study participants signed a written consent and there was no obligation for them to participate in the study. Also no one paid any charges for consultative interventions. Eight 2-hour sessions were provided for a period of 2 months. The intervention group’s participants took part in 8 sessions of group hope therapy including:

Distribution of the questionnaires and explaining the intervention (1 session).

Hope navigation: description of hope and its positive outcomes, expression of the outcomes of optimism and its role in mental health as well as improving the quality of life (1 session).

Hope consolidation: Organization of hope components; i.e., purposefulness, seeking for components and strategies, and the effect of people's attitude toward life on hope. The study participants were asked to reveal their positive and negative emotional feelings about their problems as a written task or express themselves in the group (2 sessions).

Hope improvement: Creation of specific logical goals in life related to infertility therapeutic management and satisfying conjugal life (2 sessions).

Hope maintenance: Making attempts to achieve life goals (1 session).

Comprehensive investigations of infertility treatment, strategies and psychological techniques (1 session).

Performance of post-test and summarization of the stages in the group (1 session).

In addition, the study participants were provided with a summary of previous sessions and received SMS containing positive sentences.

In this study, data collection was done using Depression Anxiety Stress Scale (DASS) developed Lovibond. This scale consists of 21 items whose score ranges from 0 (never) to 3 (very much). DASS evaluates stress, anxiety, and depression. The DASS-21 is an instrument designed to measure 3 negative affective states of depression, anxiety, and stress. "The depression scale assessed dysphoria, hopelessness, devaluation of life, self-deprecation, lack of interest or involvement, anhedonia, and inertia. The anxiety scale evaluated autonomic arousal, situational anxiety, and subjective experience of anxious affect. The stress scale assessed difficulty relaxing, nervous arousal, and becoming easily upset or agitated, irritable, or over-reactive and impatient" (Lovibond, 1998; Lovibond & Lovibond, 1995).

“This scale consisted of a general factor of psychological distress plus orthogonal specific factors of depression, anxiety, and stress” (Henry & Crawford, 2005)

After data collection, results were compared. Descriptive statistics were used in order to determine the distribution of data. Besides, student's T-test was used to compare means between the two groups before and after intervention. Also, paired T-test was employed in order to assess means within the two groups before and after intervention. Moreover, the mutual effects of demographic characteristics, hope therapy, and research tests were evaluated using ANCOVA.

## 3. Results

According to Table 1, no significant difference was observed between the two groups regarding demographic characteristics.

**Table 1:** Descriptive statistics of the two groups

	Demographic variable	Experiment group (n=28)	Control group (n=31)	Significance
Age	<20	--	1(3.2)	0.17
	21-30	14(%50)	24(77.4)	
	31-40	13(46.4 %)	6(19.4)	
	>40	1(3.6)	---	
	total	28(100)	31(100)	
level of education	Illiterate- primary	2(7.2)	11(35.5)	0.10
	Secondary	18(64.3)	19(61.3)	
	Academic	8(28.5	1(3.2)	
	Total	28(100)	31(100)	
Duration of infertility	<5	11(40.7)	10(32.3)	0.74
	6-10	14(51.9)	18(58.1)	
	11-15	2(7.4)	1(3.2)	
	>16	- -	2(6.5)	
	Total	28(100)	31(100)	

As Table 2 shows, although the percentage of people with severe disorders had no significant change in the control group, the intensity of the disorder was lower in the experiment group after intervention.

**Table 2:** Severity of the disorder in the two groups

DASS	Group	Experiment group (n=28)	Control group (n=31)
Stress pre-test	Normal	15(53.6%)	12(38.7%)
	Mild	9(32.1%)	9(29%)
	Moderate	4(14.3%)	10(32.2%)
	Severe	--	-
Stress pre-test	Normal	25(89.3)	9(29%)
	Mild	2(7.1)	8(25.9)
	Moderate	1(3.6)	10(32.2%)
	Severe	--	4(12.9)
	Total	28(100)	31(100)
Anxiety pre-test	Normal	8(28.6)	9(29)
	Mild	3(10.7)	1(3.2)
	Moderate	10(35.7)	18(58.1)
	Severe	6(21.4)	3(9.7)
	Extremity sever	1(3.6)	---
Anxiety post- test	Normal	11(39.3)	7(22.4)
	Mild	9(32.1)	6(19.4)
	Moderate	8(28.6)	18(58.1)
	Severe	-	--
	Total	28(100)	31(100)
Depression pre-test	Normal	11(39.3)	8(25.8)
	Mild	9(32.1)	16(51.6)
	Moderate	8(28.6)	7(22.6)
Depression post-test	Normal	21(75)	12(38.7)
	Mild	7(25)	15(48.4)
	Moderate	---	42(12.9)
	Total	28(100)	31(100)

**Table 3:** Mean and standard deviation of research variables between groups

DASS	Group	Mean (SD)	T
Stress pre	Experiment	12.60 (5.38)	14.68^€^
	Control	11.22(2.91)	
Stress post	Experiment	8.21(4.74)	9.1^€^
	Control	10.32(2.91)	
Depression pre-test	Experiment	10.25(2.42)	8.99^€^
	Control	11.09(3.41)	
Depression post test	Experiment	5.92(4.17)	4.33*
	Control	9.90(2.95)	
Anxiety pre	Experiment	103.5(6.01)	8.1^€^
	Control	9.70(3.81)	
Anxiety post	Experiment	6.28(5.1)	10.59*
	Control	9.83(3.12)	
DASS1	Experiment	33(15.99)	12.37^€^
	Control	32(8.95)	
DASS2	Experiment	20.42(12.05)	15.25*
	Control	29.06(7.71)	

* p<0.05(significant)

€ NS (Not significant), T(Student T- test)

Although no significant difference was observed between the two groups regarding mean differences before intervention, results revealed a significant difference in this regard after intervention.

Results of paired T-test showed no significant difference between pre-test and post-test of DASS in the control group. On the other hand, a significant difference was observed in the intervention group in this regard (Table 4).

**Table 4:** Mean and standard deviation of research variables within groups from DASS test

DASS	Group	Mean (SD)	T
Stress 1	Control	11.22(2.91)	1.41
Stress 2		10.32(2.91)	
Depression 1	Control	11.09(3.41)	1.95
Depression 2		90.90(2.95)	
Anxiety 1	Control	9.70(2.81)	0.19
Anxiety 2		9.83(3.1)	
Stress 1	Experiment	12.60(5.38)	4.61*
Stress 2		8.21(4.74)	
Depression 1	Experiment	10.35(6.01)	3.97*
Depression 2		6.28(5.1)	
Anxiety 1	Experiment	10.25(5.42)	5.57*
Anxiety 2		5.92(4.17)	
DASS1	Experiment	32.21(15.99)	5.53*
DASS2		20.42(12.65)	

* p<.0 05(significant), T= Results from (Paired T –test)

**Table 5:** The results of ANCOVA test regarding the differences between the two groups

Variable	Source	Sum of score	DF	Mean square	F
Hope therapy	Duration of infertility	114.88	3	38.29	0.34
	Age	3.429	1	3.429	.031
	Education	334.76	3	111.56	1.04
	group	1097.20	1	1097.20	10.23*

* P<0.05

Results of ANCOVA test showed that regardless of demographic variables, the mean differences between the study groups were resulted from making intervention.

Other experimental results also showed that fertility rate after a 6-month follow up was 3.33% in the experiment group while it was 0% in the control group. An intervention group participant’s pregnancy in the seventh session was an interesting result of this study. This result was confirmed by a gynecologist through diagnostic test.

## 4. Discussion

The present study aimed to investigate the effect of hope therapy on psychological problems (anxiety, depression, and stress) among infertile women under treatment by assisted reproductive techniques.

The study results showed that the treatment plan was effective in reducing depression in the intervention group, which is in agreement with the results of other studies on hope therapy. For instance, researchers performed hope-based interventions for adults suffering from depression and showed that the treatment was effective in reducing people's depression symptoms and in increasing their hope (Schmidt & Sejbæk, 2012; Klausner et al., 2002; Cheavens et al., 2006; Cheavens, 2001; Hankins, 2004). Others assessed the effectiveness of group hope therapy in increasing female students' hope and mental health. The results of that study revealed the effectiveness of the treatment program in increasing subjects' hope and reducing the symptoms of impaired social interaction efficiency and depression (Alaeddini, Kajbaf, & Molavi, 2008).

Furthermore, some researchers have indicated a negative relationship between depression and hope (Snyder, Cheavens, & Sympson, 1997; Chang & Disimone, 2001; Wells, 2005). Mehmet & Rozien (2009) reported that hope was accompanied by reduction of depression symptoms and could be taught. Moreover, positive psychotherapy not only reduced negative symptoms, but also led to change in resilience through creation of positive emotions and empowerment of character and sense.

Findings of the current study showed that intervention was highly effective in reducing DASS indexes, which is consistent with the results obtained by others. These researchers also showed that the intervention resulted in reduction of anxiety (Cheavens, 2006; Cheavens, 2001; Wells, 2005). Other results agree with our study, emphasized the effect of psychiatric intervention on psychiatric symptoms in couples with infertility.

This treatment program reduced depression, anxiety, and stress by describing hope and its positive outcomes, creating specific goals in life, and making attempt for achieving one's aims in life. In fact, having a goal gives meaning to one's life and puts one in a specific path. Besides, hope leads one to become active which helps activate other people, too. Hope can also result in self-confidence, tranquility, energy for planning and working, conformity to conditions and a good feeling (Feldman & Snyder, 2005).

Hope therapy helps people to consider and categorize definite goals, select various ways to achieve their goals, identify barriers and consider related challenges to be overcome (Clarke, 2003; Irving et al., 1997; Wilson et al., 2010; Chang & Disimone, 2001). Su, Tsann-Juu and Yueh-Chih Chen (2006) stated that hope was one of the main effective factors in successful IVF. Hopefulness plays a critical role in the improvement of various psychological disorders. In fact, improving the meaning of life leads to hopefulness and prevents psychological disorders, such as depression. It is also an important factor in individual as well as group consultation and psychotherapy (Snyder & Rand, 2005). These studies confirm our results about the effect of intervention on decreasing psychiatric symptoms among infertile people. Also using psychiatric interventions may affect successful outcomes in future treatments.

Snyder (1995) believed that hopelessness caused physical as well as mental disorders. On the other hand, hope therapy can improve the patient's mental health and quality of life. It is also highly effective in treatment of physical and mental disorders in treatment centers for couples with infertility. Findings of the present study confirmed the results of previous studies. Other psychiatric approaches on infertile couples confirmed psychological effects of this approach on mental health and improving the quality of life in couples with infertility (Noorbala et al., 2008; Mosalanejad & Khodabakhshi Koolaee, 2013; Mosalanejad, 2012; Mosalanejad, Khodabakshi Koolaee, & Shoyokh, 2012). All the above results confirm our results about the effect of psychiatric interventions on decreasing psychiatric symptoms in couple with infertility. However, more research is needed to investigate the impact of this approach on other aspects of infertility.

Research limitations include individuals have less tendency to participate in regular sessions and low number of patients with unexplained infertility, as well as other problems.

## 4. Conclusion

Overall, it can be concluded that hope increasing interventions are effective in increasing the infertile women's hope. Besides, hope therapy as a positive psychological technique can improve these women's general health and subsequently improve the family’s health. Therefore, in addition to assisted reproductive techniques, hope therapy is recommended to be presented to infertile people in order to improve their life quality and help them adapt with their problems
